# Endoscopic soft palate augmentation using injectable materials in dogs to ameliorate velopharyngeal insufficiency

**DOI:** 10.1371/journal.pone.0238646

**Published:** 2020-09-04

**Authors:** Emiko Tanaka Isomura, Makoto Matsukawa, Kiyoko Nakagawa, Ryo Mitsui, Mikihiko Kogo

**Affiliations:** First Department of Oral and Maxillofacial Surgery, Osaka University, Graduate School of Dentistry, Suita City, Osaka, Japan; Stanford University School of Medicine, UNITED STATES

## Abstract

**Background:**

Velopharyngeal structure augmentation methods are used as alternatives to pharyngeal flap operations. Recently, we investigated the sites of velopharyngeal structure augmentation in dogs and reported that the most effective injection location is the soft palate. However, there have been no reports regarding the optimal materials for implantation or injection. In this study, we aimed to investigate the injectable materials used in soft palate augmentation in dogs to ameliorate velopharyngeal insufficiency (VPI).

**Methods:**

Endoscopic soft palate augmentation (ESPA) was performed in dogs using purified sodium hyaluronate, atelocollagen, or autogenic fat tissue. ESPA is an original technique developed by our group, and this is the first report of its performance. Moreover, we assessed the amount of nasal air leakage during inspiration at rest and during expiration under the rebreathing system at 1, 2, 3, 4, 5, and 6 months after injection of these materials.

**Results:**

The amount of nasal air leakage during expiration under the rebreathing system was significantly decreased in all dogs injected with the ESPA materials, but neither apnea nor hypopnea was observed.

**Conclusions:**

We investigated the optimal materials for use in ESPA, such as purified sodium hyaluronate, atelocollagen, or autogenic fat tissue. We found that all of them reduced nasal air leakage and only autogenic fat tissue showed significant histologic differences in dogs at 6 months. This technique may also be useful for the treatment of patients with VPI.

## Introduction

When treating patients with cleft palate, velopharyngeal insufficiency (VPI) can sometimes occur after palatoplasty. VPI is the failure of the nose and mouth to separate during speech because of an anatomical dysfunction of the soft palate. Many cases of VPI are due to shortfall, poor movement of the soft palate caused by scarring, or poor reconstruction of the muscles of the soft palate. Furthermore, patients with 22q11.2 deletion syndrome have VPI due to inadequate soft palate muscle formation.

In our hospital, speech therapy is the first step in the treatment of VPI. If VPI cannot be managed with speech therapy, a speech aid is used for closure of the nasopharynx by lifting the soft palate or filling the gap. Then, after VPI is shown to be controlled with the speech aid, pharyngeal flap surgery is performed to wean the patient off the speech aid [1–3]. However, it is difficult to apply this treatment in children because it causes fundamental changes to the velopharyngeal form, which may result in sleep apnea or inability to perform nasal intubation during future orthodontic surgeries [4–7].

Several reports have described another method for treating VPI [8–22]. Velopharyngeal structure augmentation is an alternative to pharyngeal flap surgery that utilizes an injectable material implanted into the tissue around the velopharynx. However, it is not yet a standard treatment because it has not been extensively studied. Anatomic sites and injection materials vary widely, owing to the lack of standardized criteria, and their effects also differ among institutions.

Recently, we investigated the sites of velopharyngeal structure augmentation in dogs and reported that the most effective injection location is the soft palate, rather than the posterior pharyngeal wall or bilateral pharyngeal walls [23]. Dogs’ velopharynx exhibit inherently like VPI; thus, the rhinopharynx is not completely closed, even when the soft palate is lifted [24]. We injected saline intraorally, in 1-mL increments, into the nasal side of the soft palate, posterior pharyngeal wall, or bilateral pharyngeal walls of each dog. The soft palate that was injected with saline achieved steady augmentation, and nasal air leakage disappeared following the 5-mL saline injection. Conversely, nasal air leakage persisted in the dogs with saline injected in the posterior pharyngeal wall or bilateral pharyngeal walls.

There have been no reports about the optimal materials for implantation or injection, however. There are various artificial and biological materials that may be used in the velopharyngeal structure, including silicone, Teflon, porous polyethylene, Gore-Tex^®^, calcium hydroxyapatite, auricular or costal cartilage, and autologous fat [8–22]. However, when we augment the soft palate, the material needs to be injectable, because it cannot be implanted into the nasal side of the soft palate without damaging the levator veli palatini.

In this study, we aimed to investigate injectable materials for soft palate augmentation in dogs for treatment of VPI. Furthermore, we sought to introduce the endoscopic soft palate augmentation (ESPA) technique, because it causes less damage to the levator veli palatini than other techniques. ESPA is an original technique developed by us, and this is the first report of its performance. If the materials can keep the volume after injection, this technique may also be useful for the treatment of patients with VPI.

## Materials and methods

ESPA was performed at the Large Animal Laboratory of the Graduate School of Dentistry at Osaka University using 11 beagles (TOYO beagle; Oriental Yeast Co., Tokyo, Japan), aged 20–24 months and weighing 9–12 kg. All dogs were housed in separate cages and were provided solid food (Oriental Yeast Co., Tokyo, Japan) and water *ad libitum*. All experimental protocols were reviewed and approved by the Intramural Animal Care and Use Committee of Osaka University Graduate School of Dentistry (approval number: 29-004-0).

All procedures were performed under general anesthesia administered via an intramuscular injection of medetomidine (0.02 mg/kg) and midazolam (0.3 mg/kg), followed by an intraperitoneal injection of sodium pentobarbital (25 mg/kg) 15 minutes later. Animals were fixed in the supine position after the ventilation tube was passed through the mouth, and all efforts were made to minimize suffering.

**Fig 1:**
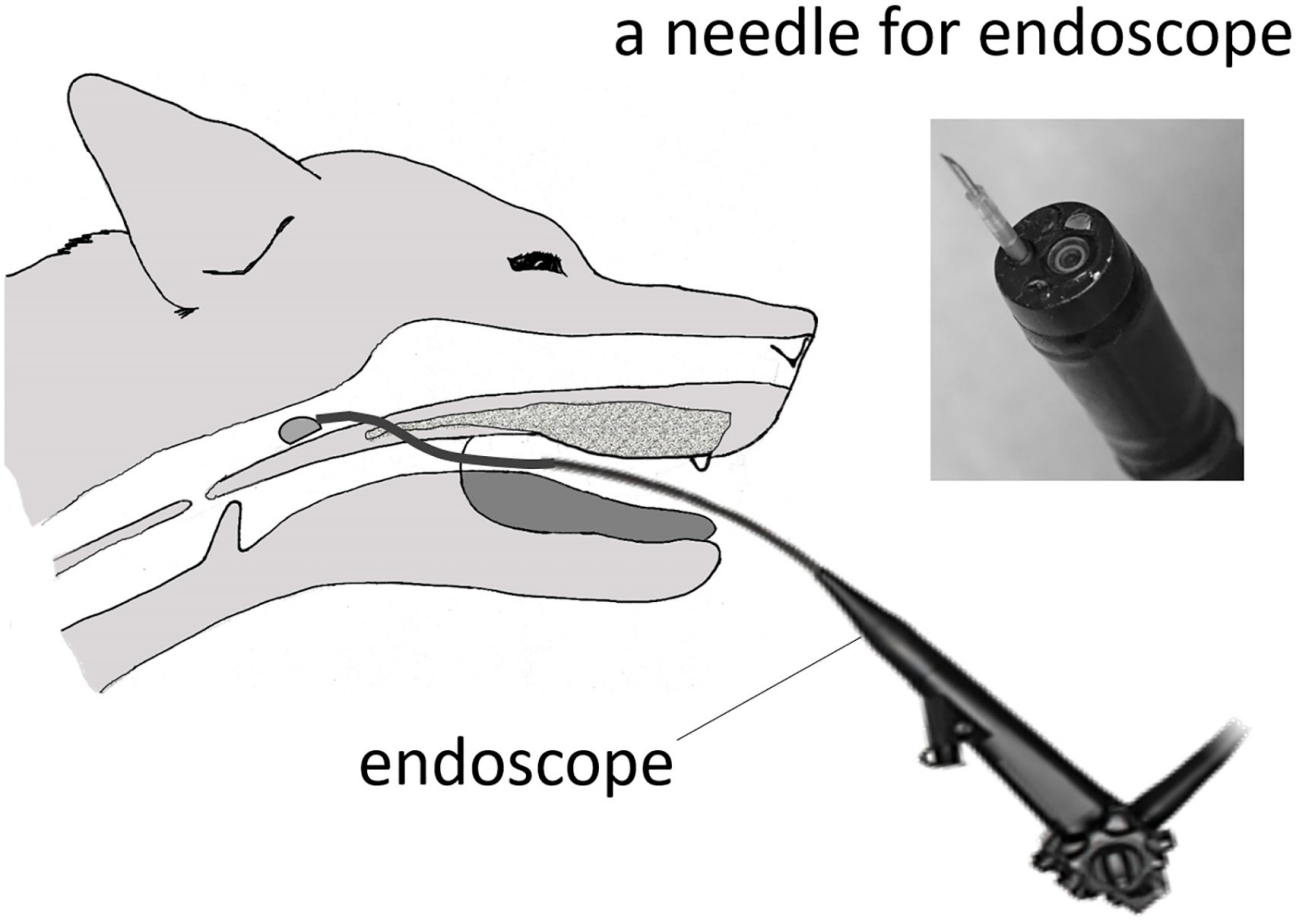
The schema of endoscopic soft palate augmentation (ESPA). An approximately 8-mm hole was created in the most anterior part of the soft palate using an electric knife to insert the endoscope. This hole needed because the endoscope could not insert by nasal approach in dog, due to narrowness of the canine nasal cavity. Then, the endoscope was inserted to the nasal side of the soft palate.

Using an electric knife, an approximately 8-mm hole was made in the most anterior part of the soft palate in each dog. This hole was needed because the endoscope cannot be inserted nasally in dogs, due to narrowness of the canine nasal cavity. The endoscope (i-Vets 8.0; SCETI K., Tokyo, Japan) was then inserted into the nasal side of the soft palate, and purified sodium hyaluronate (Hyaluronate Na^®^, Sawai Pharmaceutical Co., Ltd. Osaka, Japan, n = 3), atelocollagen (Koken Atelocollagen implant^®^, Koken Co., Ltd. Tokyo, Japan, n = 3), or autogenic fat tissue (n = 4) was injected into the nasal mucosal side of the anterior two-thirds of the soft palate using a 23-G needle (Interject™, Boston Scientific, Natick, USA) under endoscopic guidance to directly confirm entrance into the nasal mucosa (Fig 1).

**Fig 2:**
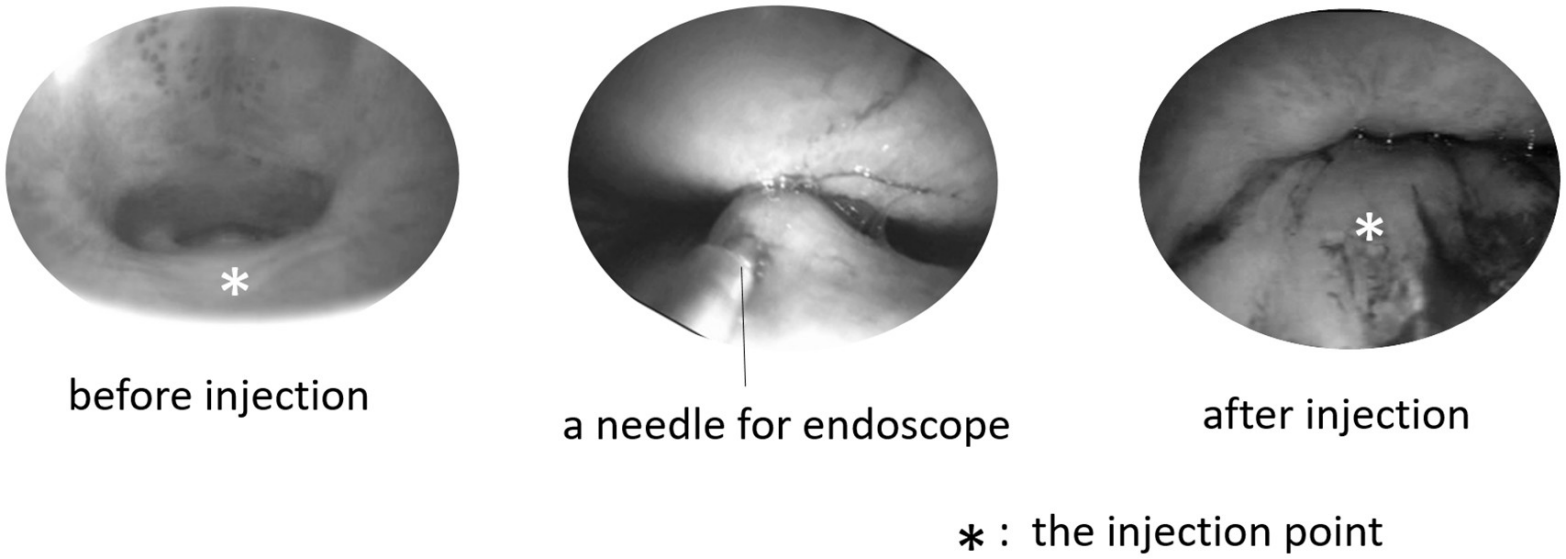
Endoscopic image during ESPA. Approximately 2-ml of the materials was injected to each of the dogs until the soft palate slightly touched the post-pharyngeal wall.

Autogenic fat tissue was taken from the greater omentum and refined using the Coleman method [25]. Vascular tissue was removed visually from the extracted greater omentum and centrifuged (3000 rpm, 3 minutes) to separate the three layers (upper layer: oil from crushed fat cell, middle layer: fat cells, bottom layer: blood, water, and lidocaine used as local anesthesia). Only the middle layer was used as an injection material. Approximately 2 ml of each material was injected into each dog until the soft palates slightly touched the post-pharyngeal walls (Fig 2).

We then assessed the amount of nasal air leakage during inspiration at rest and expiration under a rebreathing system prior to ESPA (= non-treated) and 1, 2, 3, 4, 5, and 6 months after injection of the materials, as described previously [23]. The tip of the ventilation tube was withdrawn from the trachea to the oral cavity to allow expiration through the nasal cavity. After removing the electrode and ventilation tube to prevent oral air leakage during measurement, the oral cavity was filled with an alginate impression material. While the dogs were under the rebreathing system, the amount of air leakage from the nasal cavity was measured by a flow meter (TSD117; BIOPAC Systems Inc., Japan) using the rubber tubes connected to the flow meter’s sensor in front of both nasal apertures. The external portion of the rubber tubes was packed with quick, self-curing acrylic resin (UNIFAST II; GC Co., Tokyo, Japan) to prevent air leakage. Data from the flow meter was recorded on a personal computer (U24a-px3210r Windows7; ASUSTek Computer Inc., Japan) using data acquisition and analysis software (Labchart7; AD Instruments, Japan) through a DC Amplifier (DA100C; BIOPAC Systems Inc., Japan), an analog output module (HLT100-C; BIOPAC Systems Inc., Japan), and an AD converter (Power lab; AD Instruments Co., Tokyo, Japan).

Data from the nasal air leakage of one breath was separated into the inspiration phase and expiration phase, and their integral value was measured. We assessed the amount of nasal air leakage during inspiration at rest to determine the presence of apnea or hypopnea and nasal air leakage during expiration under the rebreathing system to evaluate the effect of ESPA. After euthanasia at 6 months post-injection, histological examinations were performed in all dogs. The soft palates were dissected and stained with hematoxylin and eosin.

Normality of the data was evaluated and, owing to their nonparametric nature, analyzed using the Kruskal–Wallis test post-hoc Mann–Whitney’s U test (p-value < 0.05). All statistical analyses were conducted using R version 2.8.1 (CRAN: https://cran-archive.r-project.org/bin/windows/base/old/2.8.1/).

## Results

**Fig 3:**
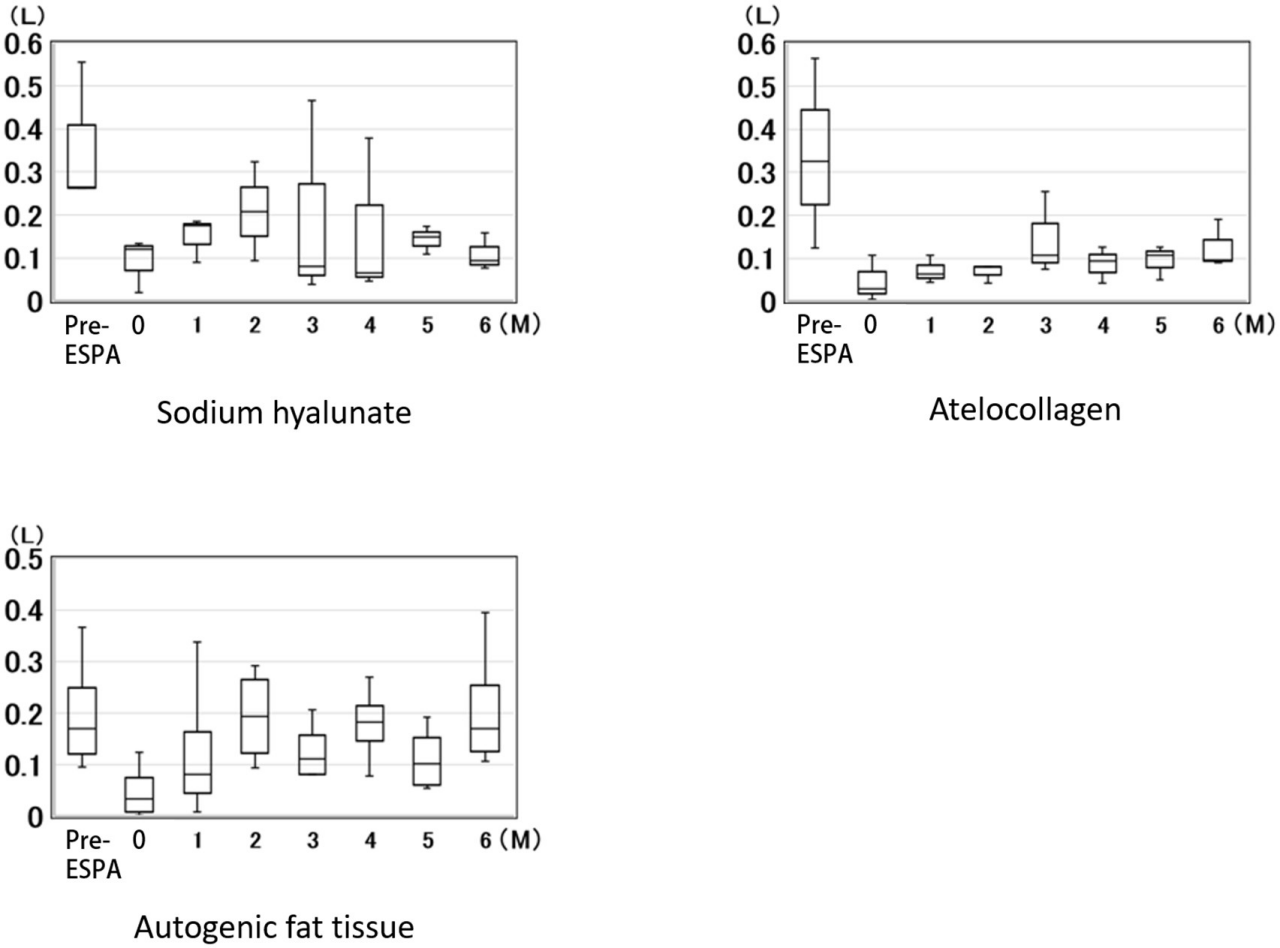
The amount of nasal air leakage during inspiration at rest. The changes of nasal air leakage during inspiration at rest (soft palate was not lifted). The amount of nasal air leakage during inspiration in each dog decreased slightly, compared to the pre-ESPA value, but the decrease was not enough to cause apnea or hypopnea. (0 = Immediately after ESPA).

**Fig 4:**
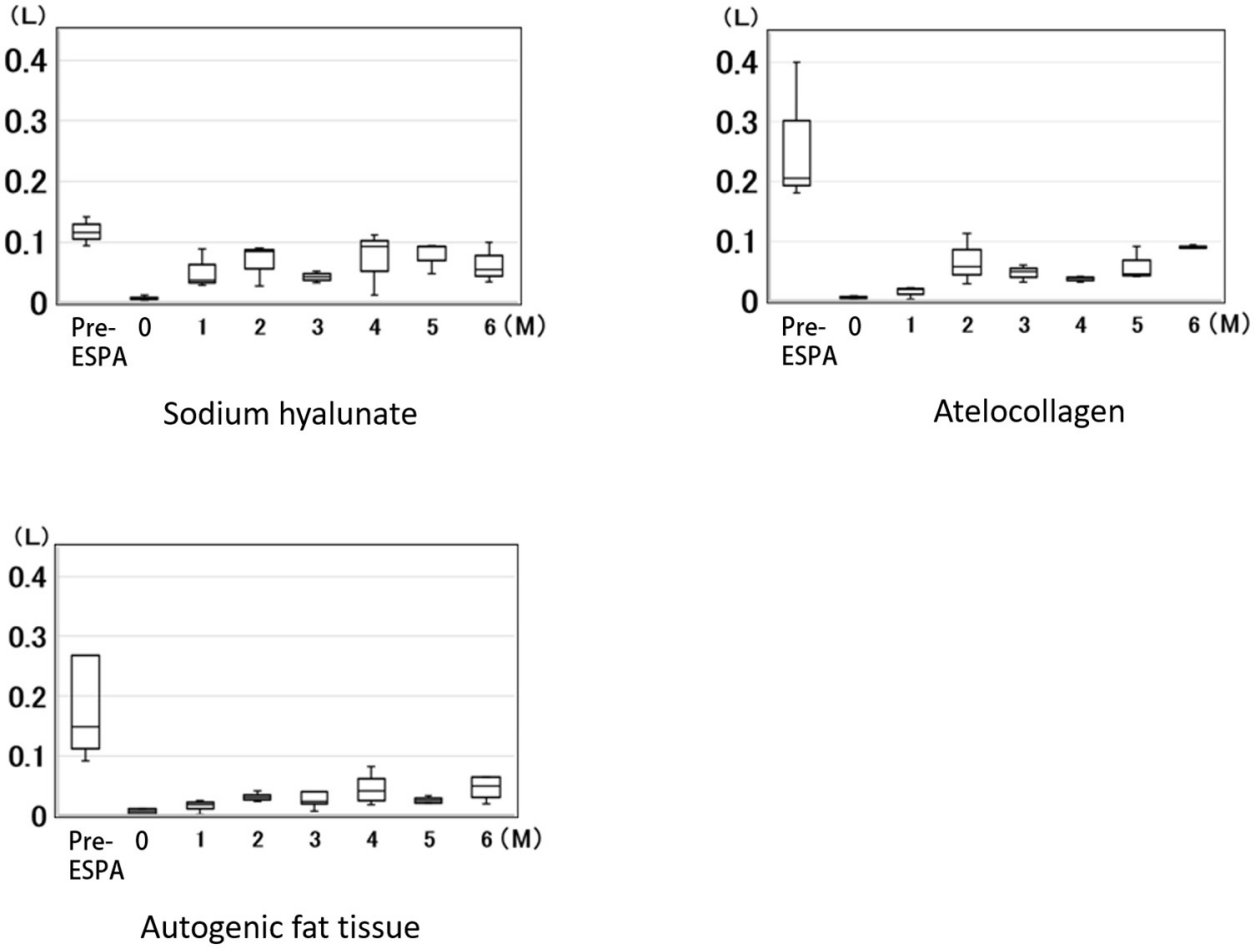
The amount of nasal air leakage during expiration under rebreathing. The changes of nasal air leakage during expiration under rebreathing (soft palate was lifted due to levator veli palatini action). The amount of nasal air leakage during expiration under the rebreathing system was significantly decreased in all dogs injected with materials used for ESPA, compared with pre-ESPA (p<0.05). (0 = Immediately after ESPA).

**Fig 5:**
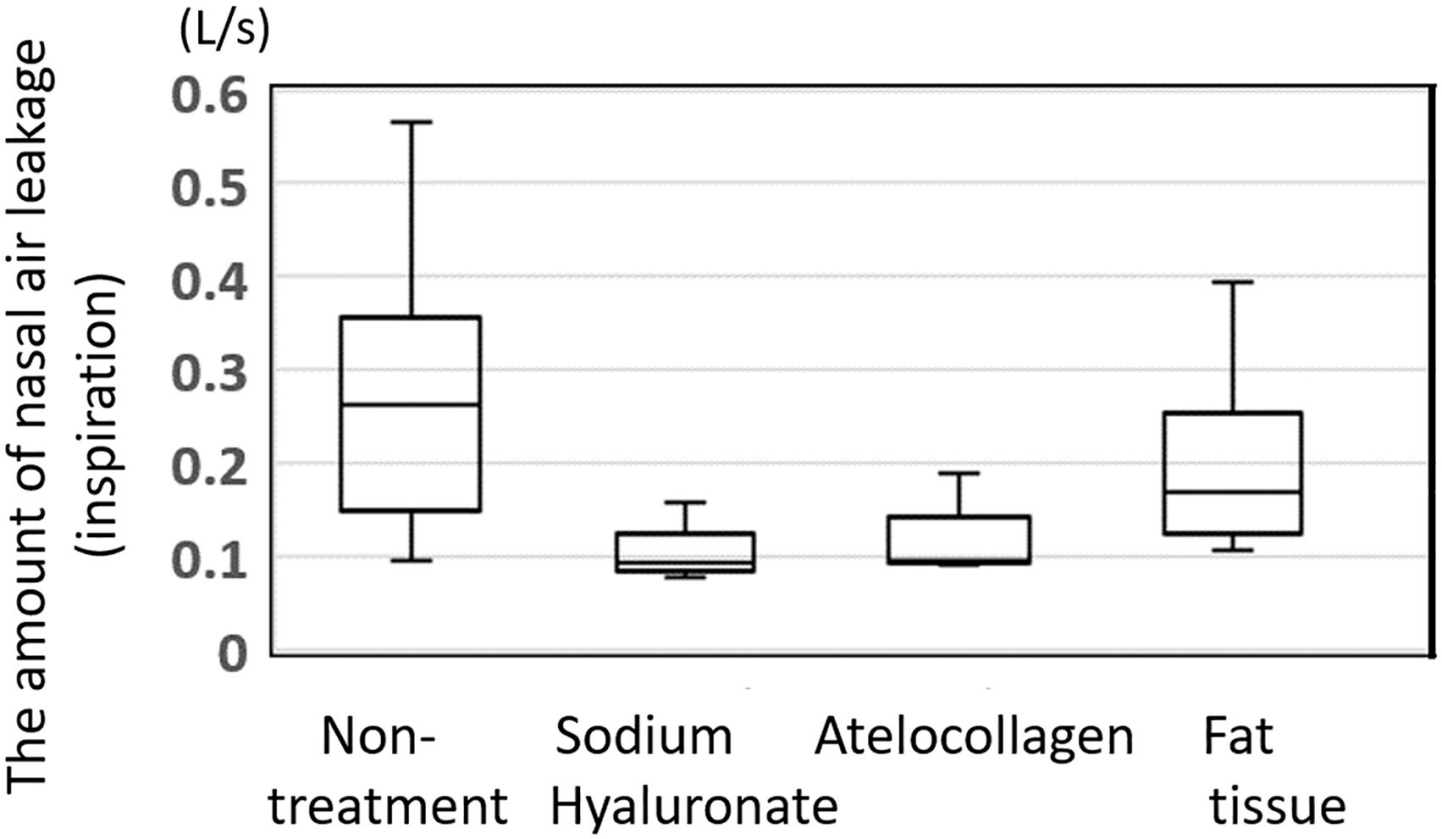
The amount of nasal air leakage during inspiration at rest. The amount of nasal air leakage during inspiration at rest decreased in all dogs compared to the pre-ESPA value, but apnea or hypopnea was not observed. Moreover, no significant difference in the outcomes was observed among the materials.

The changes of nasal air leakage, presented in medians and inter-quartile ranges that occur over time are shown in Figs 3 and 4. Fig 3 shows nasal air leakage during inspiration at rest (soft palate was not lifted), and Fig 4 shows nasal air leakage during expiration under rebreathing (soft palate was lifted due to levator veli palatini action). The amount of nasal air leakage during inspiration in each dog decreased slightly, compared to the pre-ESPA value, but the decrease was not enough to cause apnea or hypopnea (Fig 3). Comparison of data at 6 months post-injection among the three materials is shown in Fig 5, and no significant difference was found.

**Fig 6:**
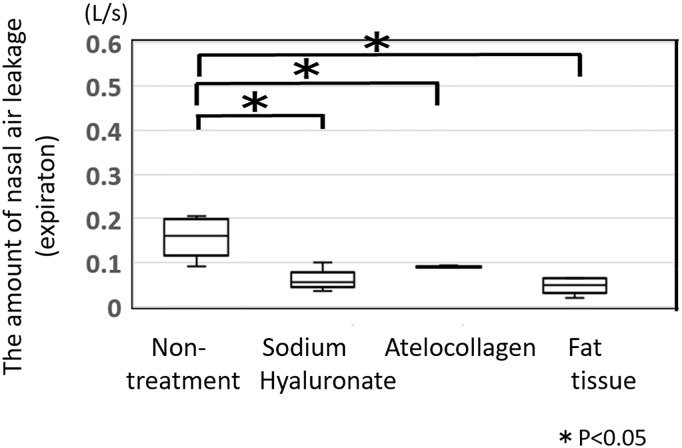
The amount of nasal air leakage during expiration under the rebreathing system. The amount of nasal air leakage during expiration under the rebreathing system decreased significantly in all the dogs injected with any of the materials during ESPA. The median amount of nasal air leakage of the non-treated dogs (pre-ESPA: n = 10) was 0.16 L/sec, whereas at 6 months after ESPA, the median amount of nasal air leakage during expiration was 0.055, 0.089, and 0.049 L/sec in dogs injected with purified sodium hyaluronate, atelocollagen, and autogenic fat tissue, respectively.

Conversely, the amount of nasal air leakage during expiration under the rebreathing system was significantly decreased in all dogs injected with materials used for ESPA, compared with pre-ESPA (p<0.05) (Fig 4). The median amount of nasal air leakage during expiration in the non-treated dogs (pre-ESPA: n = 10) was 0.16 L/sec, whereas at 6 months after ESPA, the median amount of nasal air leakage during expiration was 0.055, 0.089, and 0.049 L/sec in dogs injected with purified sodium hyaluronate, atelocollagen, and autogenic fat tissue, respectively (Fig 6).

**Fig 7:**
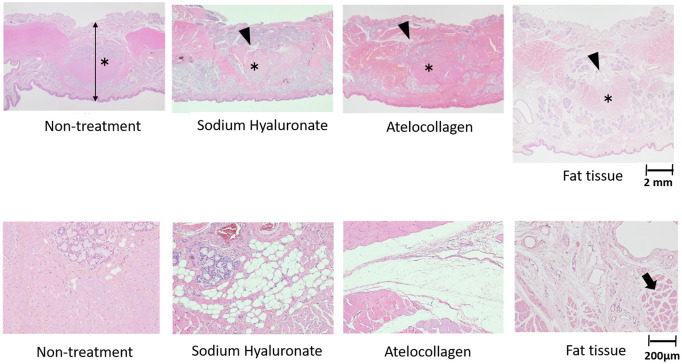
Histological results. The maximum value of the soft palate thickness between the dogs injected with sodium hyaluronate, atelocollagen and non-treated dogs was the same. Fat tissues were observed around the injection site of the soft palate of the dogs injected with purified sodium hyaluronate, whereas fibrous tissues were observed in those injected with atelocollagen. In contrast, fibrous tissues and vasculatures, appearing as lymphatic or blood vessels, with minimal muscle tissues (arrow) were observed around the injection site in the dogs injected with autogenous fat. (Asterisks are levator veli palatini).

Histologically, the maximum soft palate thickness between the dogs injected with sodium hyaluronate or atelocollagen and non-treated dogs was the same (Fig 7; Table 1). Fat tissues were observed around the soft palate injection site of the dogs injected with purified sodium hyaluronate, whereas fibrous tissues were observed in those injected with atelocollagen. In contrast, fibrous tissues and vasculatures, appearing as lymphatic or blood vessels with minimal muscle tissues, were observed around the injection site in the dogs injected with autogenous fat.

**Table 1:** The thickness of the soft palate at 6 months after the endoscopic soft palate augmentation (ESPA) (mm).

Non-treatment	Sodium hyaluronate	atelocollagen	Fat tissue
7.03 (6.95–7.63)	6.84 (6.55–6.85)	7.56 (7.45–8.19)	10.0 (8.58–11.1)

## Discussion

In this study, we investigated various materials that can be used in ESPA. ESPA is especially useful because it does not cause injury to the musculus levator veli palatine, and injectable materials can be implanted while simultaneously monitoring the volume of augmentation through an endoscope. Injection of materials intraorally has been demonstrated previously, but this method is challenging when trying to inject into the mucosal layer, as the needle may penetrate the nasal mucosa [22]. Using our technique, we had to create a hole to insert the endoscope because the device cannot pass through the nasal cavity in dogs. However, in humans it can pass through the nasal cavity; thus, creating a hole is not necessary.

We selected purified sodium hyaluronate, atelocollagen, and autogenic fat tissue as the materials to investigate because they can be injected into the soft palate through a needle. Sodium hyaluronate is a mucopolysaccharide, and its molar weight is 1,000,000 g/mol, while atelocollagen is a protein and its molar weight is 300,000 g/mol. One gram of sodium hyaluronate can hold 6,000 ml of water; hence, even if sodium hyaluronate itself is absorbed gradually, the surrounding water remains, and that makes it easy to retain the entire volume for an extended period. And all the materials reduced the amount of nasal air leakage during expiration under rebreathing. Soft palate movement was not hindered by any of the materials, and apnea or hypopnea did not occur in any of the dogs.

Yasuda investigated histological changes in the skin after injection with sodium hyaluronate or atelocollagen [26]. He reported that the bulging area where the sodium hyaluronate was injected disappeared after 60 days, and only a small amount of sodium hyaluronate remained in the site. He also reported minimal fibrous tissue was observed histologically at 180 days after injection. Conversely, the bulging area where atelocollagen was injected remained even at 180 days after injection, and the material converted to collagen. In our study, sodium hyaluronate was not observed histologically at 6 months after injection, whereas atelocollagen still remained at the 6-month follow-up, and new collagen was observed around the musculus levator veli palatini after injection, consistent with Yasuda’s findings.

Multiple studies have reported that 30%–70% of the autologous transplant of fat graft was resorbed within a year [27–30]. Guerrerosantos et al. reported that fat grafts injected intramuscularly were successful, owing to the excellent circulation of muscular tissue, compared to those injected subcutaneously [30]. In our study, we could not confirm whether the autogenic fat tissue was injected intramuscularly or subcutaneously, but histologically, no damage to the levator veli palatini by the injection was observed. Only a small amount of fat tissue remained around the muscle tissue during follow-up, and vascularization was observed histologically in the mucosa layer. Soft palate thickness was biggest in the dogs injected with autogenic fat tissue. It was unclear, however, if this observation was due to increased muscle tissue in the soft palate. By the final observation, no fat tissue remained in any of the animals, instead it was replaced by fibrous tissue. This result may indicate that the thickness of the soft palate may remain for a long period of time.

Although ESPA using purified sodium hyaluronate, atelocollagen, or autogenic fat tissue showed good results after 6 months, its long-term effects should still be considered. Several reports have described that human lipoaspirate contains multipotent cells and may represent an alternative stem cell source for bone marrow-derived mesenchymal stem cells [31, 32]. Li et al. reported that fat grafts consisting of 10^5^/ml adipose-derived stem cells constitute an ideal transplant strategy, which may result in decreased absorption and accelerated fat regeneration [33]. Adipose-derived stem cells are one of the most widely used stem cell types for the treatment of bone and cartilage disease, Crohn’s disease, heart diseases, kidney disease, neurological disease and respiratory disease, as well as for cosmetic and plastic surgery [34]. In addition, some reports describe the effectiveness of injectable adipose tissue-derived stem cells in treating stress urinary incontinence or vocal fold paralysis [35–37]. In the future, we should also investigate whether transplantation of adipose tissue- derived stem cells can help in treating VPI. If we can make the musculus levator veli palatini stronger with this method, it may be the best treatment for VPI.

This study has several limitations. The structure of the velopharynx in dogs and that in humans are quite different, and the assessment of speech cannot be performed in dogs. In dogs, the larynx is located directly behind the base of the tongue and soft palate and lies between the pharynx and trachea. The larynx covers the trachea during swallowing so that food does not enter the windpipe. However, the soft palate of dogs may lift like that of humans during respiration. VPI cannot strictly be assessed in dogs; only analogous comparisons can be made. Therefore, we eventually must conduct further investigations in humans. We plan to perform ESPA on humans in a clinical setting soon.

In a systematic review, Nigh E et al. described that autologous fat injection has been advocated for correction of mild to moderate VPI, but it was difficult to adapt in severe VPI [22]. At present, no ideal treatment is available for severe VPI. However, we have previously reported that the optimal site for injection is the nasal side of the soft palate and improvement of VPI was dependent on the amount of injected autologous fat [23]. ESPA may control the amount of fat easier than traditional methods; hence, we believe it could be adapted to treat severe VPI as well.

In conclusion, we investigated the optimal materials for use in ESPA, such as purified sodium hyaluronate, atelocollagen, or autogenic fat tissue. We found that all of them reduced nasal air leakage and only autogenic fat tissue showed significant histologic differences at 6 months in dogs. These results suggest that this technique may also be useful for the treatment of patients with VPI.
